# Advanced Dental Care: β-Chitosan Zinc Oxide Nanoparticles Targeting Cariogenic Microorganisms

**DOI:** 10.7759/cureus.66296

**Published:** 2024-08-06

**Authors:** Nishitha Rajasekar, Karthik Ganesh Mohanraj, Taniya Mary Martin, Meenakshi Sundaram K

**Affiliations:** 1 Department of Anatomy, Saveetha Dental College and Hospitals, Saveetha Institute of Medical and Technical Sciences (SIMATS) Saveetha University, Chennai, IND; 2 Department of Anatomy, Biomedical Research Unit and Laboratory Animal Center, Saveetha Dental College and Hospitals, Saveetha Institute of Medical and Technical Sciences (SIMATS) Saveetha University, Chennai, IND

**Keywords:** molecular docking, bacterial enzymes, biofilm inhibition, antimicrobial properties, dental caries, β-chitosan-derived zinc oxide nanoparticles

## Abstract

Introduction

Dental caries, primarily caused by cariogenic microorganisms, remains a significant global health concern. β-Chitosan, known for its biofilm-targeting properties, and zinc oxide (ZnO) nanoparticles (NPs), recognized for their potent antimicrobial effects, offer a promising approach for caries prevention and treatment. This study investigates the synthesis, characterization, and antimicrobial properties of β-Chitosan-derived ZnO NPs (β-Ch-ZnO-NPs) against these pathogens.

Methodology

β-Chitosan from fresh squid bones was isolated using demineralization and deproteinization methods. β-Ch-ZnO-NPs were synthesized and characterized using UV-vis spectroscopy and Fourier-transform infrared spectroscopy (FTIR) to confirm their size, shape, and stability. Antibacterial efficacy(agar well plate method)was assessed through standardized assays, demonstrating significant inhibition of cariogenic bacteria. The results were represented as mean± standard deviation. The Kruskal-Wallis test with post hoc analysis (Mann-Whitney U test) was conducted for statistical analysis. Molecular docking studies (blind docking method) were conducted to elucidate the interactions between β-Ch-ZnO-NPs and key bacterial enzymes involved in microbial genetic material synthesis, also known as dihydroorotate dehydrogenase (DHODH, PDB ID-2J0Y).

Results

The synthesized β-Ch-ZnO-NPs exhibited well-defined characteristics verified by UV-vis spectroscopy and FTIR confirming their nanoparticulate nature and stability. The antimicrobial effects of Streptomycin (50 µg/mL) and β-Ch-ZnO-NPs were compared across various microorganisms. β-Ch-ZnO-NPs at 100 µg/mL consistently showed larger inhibition zones than Streptomycin and β-Ch-ZnO-NPs at 50 µg/mL against *Escherichia coli​​, Enterococcus faecalis, Staphylococcus aureus, Streptococcus mutans, *and *Candida albicans.*This suggests that β-Ch-ZnO-NPs at a higher concentration have potent antimicrobial activity across a broad spectrum of pathogens, highlighting their potential as effective antimicrobial agents. Kruskal-Wallis test showed statistically significant differences (*P *< 0.001) for all microbes, and post hoc analysis (Mann-Whitney U test) confirmed the *P*-value was less than 0.05.* *Molecular docking studies indicated strong binding affinities between β-Ch-ZnO-NPs and bacterial enzymes crucial for biofilm formation, suggesting inhibition of enzyme activity critical for bacterial virulence and survival.

Conclusions

This study highlights the synergistic potential of β-Chitosan and zinc oxide NPs in combating dental caries. The synthesized β-Ch-ZnO-NPs demonstrated effective antimicrobial activity against cariogenic microorganisms, attributed to their ability to disrupt bacterial metabolism and inhibit biofilm formation. Molecular docking analysis provided mechanistic insights into how β-Ch-ZnO-NPs interact with bacterial enzymes, reinforcing their role in impeding biofilm development. Overall, the findings support using β-Ch-ZnO-NPs as a promising therapeutic strategy for preventing and treating dental caries, leveraging their combined biofilm-targeting capabilities and antimicrobial effects.

## Introduction

In the realm of oral health, dental caries, commonly known as tooth decay, remains a prevalent issue primarily instigated by the activities of cariogenic microbes, such as *Candida albicans, Streptococcus mutans, Enterococcus faecalis, Escherichia coli, Staphylococcus aureus *that* *colonize the oral cavity, forming biofilms on the tooth surfaces [1]. These bacteria thrive in the oral environment, fermenting dietary carbohydrates to produce acids that erode tooth enamel. Left unchecked, this acidic assault can lead to the formation of cavities and severe cases, tooth loss. Despite advancements in oral hygiene practices and therapeutic approaches, the persistent challenge of bacterial resistance to conventional antimicrobial agents necessitates the exploration of novel strategies for preventing and treating dental caries [2]. Among the promising avenues of research lies the application of nanoparticles (NPs), which have garnered considerable attention due to their unique physicochemical properties and potential therapeutic benefits in dentistry. Specifically, β-Chitosan-derived zinc oxide (ZnO) NPs represent a burgeoning area of interest. β-Chitosan, derived from chitin offers several advantageous properties including biocompatibility, biodegradability, and inherent antimicrobial activity, making it an ideal candidate for incorporation into NP formulations [3,4]. Complementarily, ZnO NPs are recognized for their robust antimicrobial efficacy against a broad spectrum of microorganisms. This efficacy stems from their ability to generate reactive oxygen species (ROS) upon exposure to ultraviolet light, which exerts oxidative stress on microbial cells, thereby impairing their viability [5].

The synergy between β-Chitosan and ZnO NPs has been explored to capitalize on their respective strengths in combating dental caries. β-Chitosan enhances the targeting of bacterial biofilms - a critical factor in caries progression - while ZnO NPs contribute potent antimicrobial actions, potentially surpassing the limitations of traditional therapies. By combining these components, researchers aim to develop novel approaches that effectively disrupt bacterial biofilms and inhibit microbial growth within the oral cavity [6,7]. The synthesis of β-Chitosan-derived ZnO NPs (β-Ch-ZnO-NPs) involves various methodologies aimed at achieving precise control over particle size, shape, and stability crucial for their biomedical applications. Techniques such as UV-vis spectroscopy and Fourier-transform infrared spectroscopy (FTIR) are routinely employed to characterize these NPs, confirming their structural integrity, surface functional groups, and stability in diverse environments [8]. The size and morphology of the NPs play pivotal roles in determining their antimicrobial efficacy, with smaller particles possessing greater surface area for enhanced interaction with bacterial cells and biofilms. Beyond their antimicrobial properties, β-Ch-ZnO-NPs demonstrate biocompatibility enhancements attributed to β-Chitosan, mitigating cytotoxic effects and enhancing compatibility with biological systems. This attribute is pivotal for their prospective use in medical implants, drug delivery systems, and tissue engineering applications aimed at oral health interventions. Functionalization of β-Chitosan ZnO NPs with targeting ligands or imaging agents holds promise for precise therapeutic delivery and diagnostic imaging. Such modification enables the NPs to selectively bind to specific cells or tissues, facilitating targeted treatments while enabling non-invasive visualization of disease sites - a critical advantage in dental and medical contexts alike. Research efforts are actively focused on comprehending the long-term impacts and biodegradability of β-Ch-ZnO-NPs to ensure their safety for long-term impacts and biodegradability of β-Ch-ZnO-NPs to ensure safety for human use and minimal environmental impact. Regulatory frameworks are concurrently involved to govern their incorporation into consumer products and medical devices, ensuring adherence to stringent safety standards. Looking forward, future research directions aim to optimize synthesis methodologies to scale up production while maintaining the quality and consistency of β-Chitosan ZnO NPs. Additionally exploring novel applications - ranging from cancer therapy and neural regeneration to environmental monitoring promises to expand the scope of these NPs beyond their current dental care applications. In summary, β-Ch-ZnO-NPs represent a multifaceted innovation in the realm of oral health, offering a potent arsenal against cariogenic microorganisms through the ability to penetrate biofilms, disrupt cellular membranes, and induce oxidative stress [9]. This holistic approach targets bacteria and those within biofilms, addressing the complex pathogenesis of dental caries and paving the way for advanced therapeutic strategies in oral health care [10,11].

## Materials and methods

Synthesis of β-Ch-ZnO-NPs

β-Chitosan was isolated from squid with slight modification by adapting the previous method [11]. Squid bones were washed and dried in sunlight for four days, then ground and sieved to 80 mesh. Fifty grams of the powder were demineralized in a 1 M hydrochloric acid solution (1:10) for three hours, then filtered and washed until chloride ions were removed. The residue was well treated with 1 M sodium hydroxide (1:10), heated at 60^◦^C, filtered, and washed until neutral. The resulting chitosan was derived at 70 ^◦^C and stored at -80 ^◦^C. To synthesize β-Ch-ZnO-NPs, a zinc ion solution was prepared by dissolving 0.1 mM of zinc salt. Additionally, a β-chitosan solution was also prepared These solutions were then mixed under constant stirring to ensure thorough homogenization [12]. Subsequently, a freshly prepared 0.1 M sodium borohydride solution was added dropwise to the mixture while vigorously stirring to initiate the reduction of zinc ions, leading to the formation of β-Chitosan NPs. Stirring was continued for 30 minutes to complete the reduction process and stabilize the NPs. The resulting NP solution was then centrifuged at 10,000 rpm for 20 minutes to separate β-Ch-ZnO-NPs. Stirring was continued for 30 minutes to complete the reduction process and stabilize the NPs. The resulting NP solution was then centrifuged at 10,000 rpm for 20 minutes to separate the β-Chitosan ZnO NPs from any unreacted materials and by-products. After discarding the supernatant, the NPs underwent multiple washes with deionized water to eliminate residual reactants, ensuring the purity and stability of the synthesized β-Ch-ZnO-NPs [13].

Characterization of β-Ch-ZnO-NPs

Following the synthesis of β-Ch-ZnO-NPs, characterization involved several analytical techniques. UV-vis spectroscopy was employed to scan the NPs, detecting any absorbance changes within the wavelength range of 200-700 nm. The particle size of β-Ch-ZnO-NPs was calculated using the Debye-Scherrer equation, where λ represents the X-ray wavelength, β is the full width at half maximum (FWHM), and θ is the Bragg’s angle. Fourier transform infrared spectroscopy (FTIR) using KBr pellets in the 500-4,000 cm⁻¹ range identified functional groups present in the β-Chitosan extract responsible for reducing zinc ions to NPs. These characterization techniques collectively provided comprehensive insights into the structural, morphological, and chemical properties of β-Ch-ZnO-NPs [14].

Evaluation of antimicrobial efficacy by antimicrobial assay

Using a disc diffusion assay, the antimicrobial efficacy of β-Ch-ZnO-NPs was evaluated against *Candida albicans, S. mutans, Enterococcus faecalis, Escherichia coli, *and* Staphylococcus aureus* bacterial strains. Bacterial strains were cultured in Lysogeny Broth (LB )at 37 °C for 24 hours and subsequently spread onto LB agar plates to obtain bacterial suspensions [15]. Fungi were cultured on potato dextrose agar at 25 °C in darkness. Suspensions containing approximately 1 × 10^6^ colony-forming units (CFU) of each microorganism were spread on LB or potato dextrose (PD) agar plates using a sterilized glass spreader. Sterile filter paper discs (6 mm diameter) were loaded with a fixed concentration of β-Ch-ZnO-NPs, while sterile water served as the negative control and standard antibiotics as the positive controls. Plates were then incubated at 37 °C for 24 hours. After incubation, the diameter of the inhibitory zones formed around the discs loaded with different concentrations of β-Ch-ZnO-NPs was measured to assess their antimicrobial activity. All experiments were performed in triplicate to ensure the reliability and reproducibility of the results [16].

Molecular docking studies

A molecular docking study (Blind docking method) was analyzed by using the AutoDock software (version 4.2) to explore the interaction between β-Ch-ZnO-NPs and the protein receptor β-Chitosan, sourced from the RCSB Protein Data Bank (PDB:2JOY), which is integral to bacterial microbial DNA biosynthesis. Before the submission, the 2JOY receptor was prepared by assigning Gasteiger partial charges and adding polar hydrogen atoms. The Lamarckian genetic algorithm guided the docking process, with autogrid parameters tailored to generate a comprehensive grid map covering the entire surface of the 2JOY protein. The docking simulations aimed to ascertain the optimal binding mode and sites of interaction between β-Ch-ZnO-NPs and 2JOY. The pose exhibiting the most negative binding energy was selected as the best-docked model and subsequently analyzed to visualize binding interactions and sites using BIOVIA software. This approach yielded insights into how β-Ch-ZnO-NPs interact with 2JOY, potentially influencing bacterial fatty acid metabolism [17].

## Results

β-Ch-ZnO-NPs were synthesized using a method involving the reduction of zinc ions by β-Chitosan, resulting in distinctive physicochemical properties. β-Chitosan is known for its biocompatibility and antimicrobial activity, making it suitable for incorporation into NPs. The synthesis process of β-Ch-ZnO-NPs combines the antimicrobial efficacy of ZnO NPs with β-Chitosan's biofilm-targeting capabilities, potentially enhancing their effectiveness against cariogenic microorganisms such as *C. albicans, S. mutans, E. faecalis, E. coli, *and* S. aureus. *Characterization studies using UV-vis spectroscopy confirmed the formation of β-Ch-ZnO-NPs, showing absorbance peaks characteristic of ZnO NPs. 

UV-vis spectroscopy analysis

**Figure 1:**
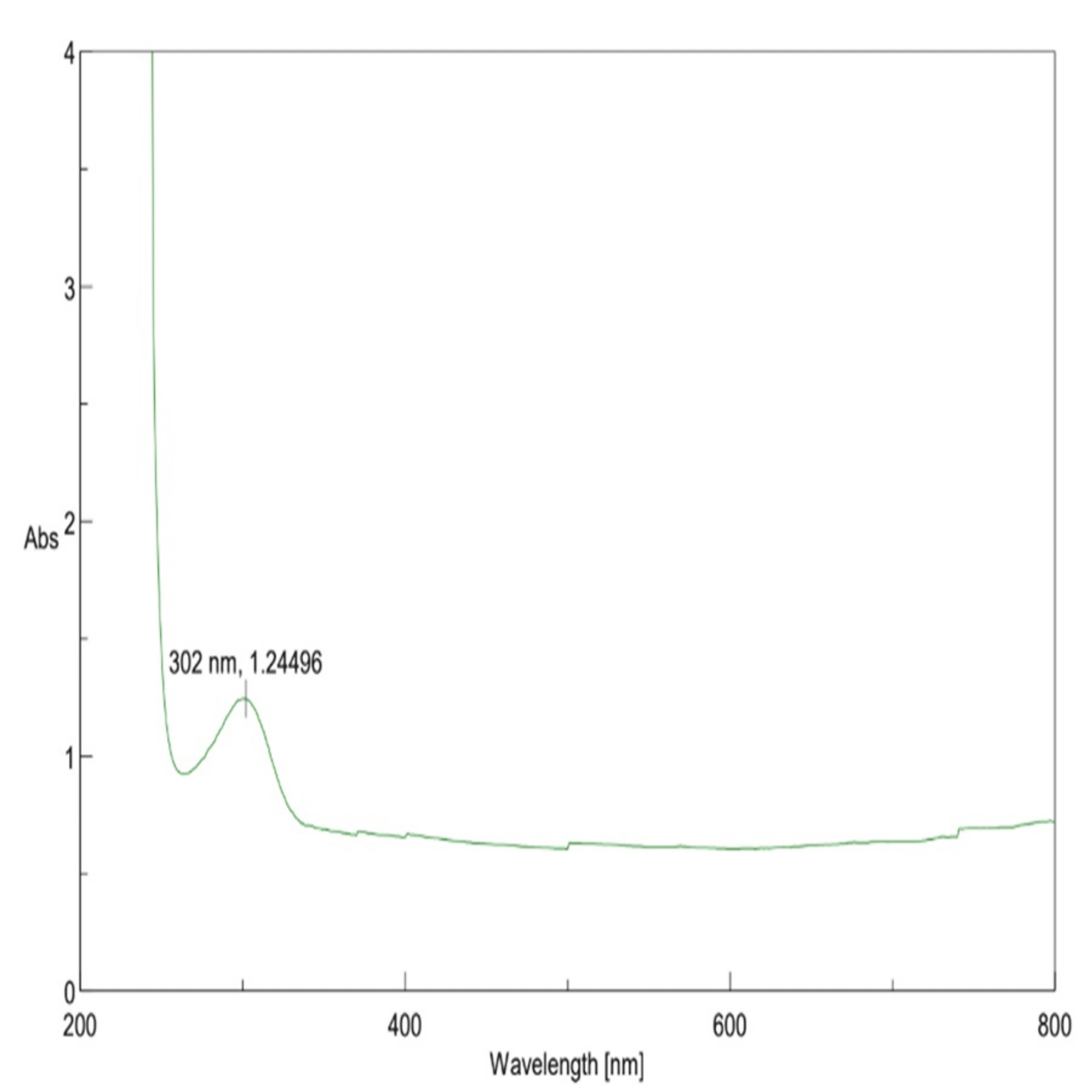
UV-vis absorption spectra of β-Chitosan-derived zinc oxide nanoparticles.

Biogenic β-Ch-ZnO-NPs were characterized using UV-visible spectroscopy, revealing a distinct exciton band at 377 nm. This absorption peak closely matches the bulk exciton absorption of β-Ch-ZnO-NPs (373 nm), indicating the formation of spherical NPs with an average size range of 40-60 nm. The rapid increase in absorbance upon excitation from the NP's ground state to its excited state further verifies its optical properties (Figure 1).

FTIR analysis

**Figure 2:**
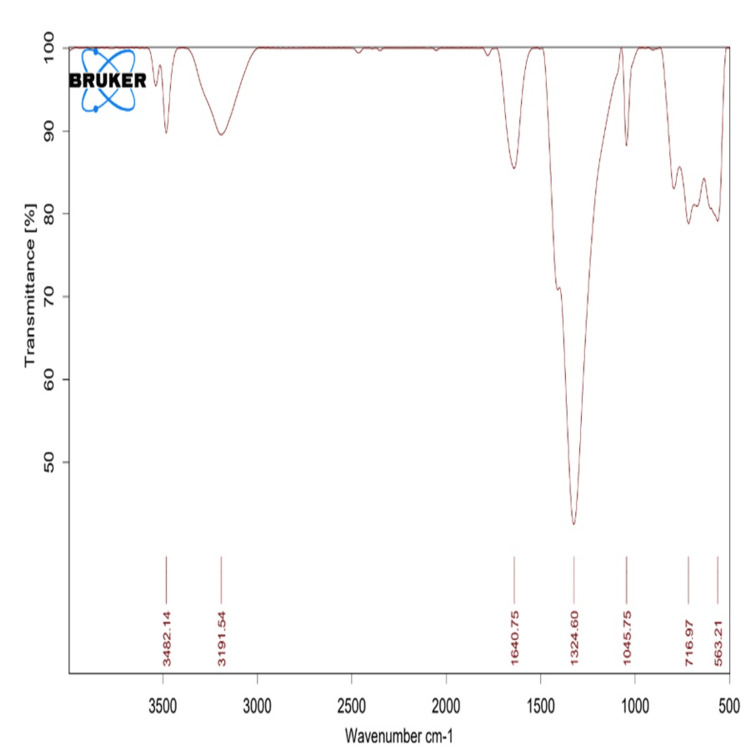
FTIR spectra of β-Chitosan-derived zinc oxide nanoparticles. FTIR, Fourier transform infrared spectroscopy

The FTIR analysis of biosynthesized β-Ch-ZnO-NPs was employed to confirm putative functional groups present in the extract and their involvement in the reduction of Zn2+ to Zn0, as well as in capping and stabilizing the bio-reduced NPs. As depicted in Figure 2 of the IR spectrum, a prominent peak at 3,371 cm⁻¹ corresponds to the O-H stretching vibration of the alcohol functionality. In the β-Ch-ZnO-NPs spectrum, a weaker peak is observed around 3,400 cm⁻¹ compared to the extract's FTIR spectrum. This suggests that bioactive compounds containing OH groups are involved in the synthesis of β-Ch-ZnO-NPs. Additionally, informative peaks at 2,890 cm^−1^ and a slightly split peak at 1,639 cm^−1^ corresponded to the stretching vibrations of C-H bonds and C═C fused with C═O bonds of alkane groups and ketones, respectively. The significant peak at approximately 499 cm^−1 ^in the FTIR spectrum of β-Ch-ZnO-NPs, indicative of metal-oxygen (M-O) bonding, further supports the formation of nanoparticles. Spectral analysis of the extract indicated the presence of phytochemicals such as phenols, terpenes, and flavonoids, which likely play a crucial role in the reduction of metal ions to form β-Ch-ZnO-NPs.

Antimicrobial potential of β-Ch-ZnO-NPs

**Figure 3:**
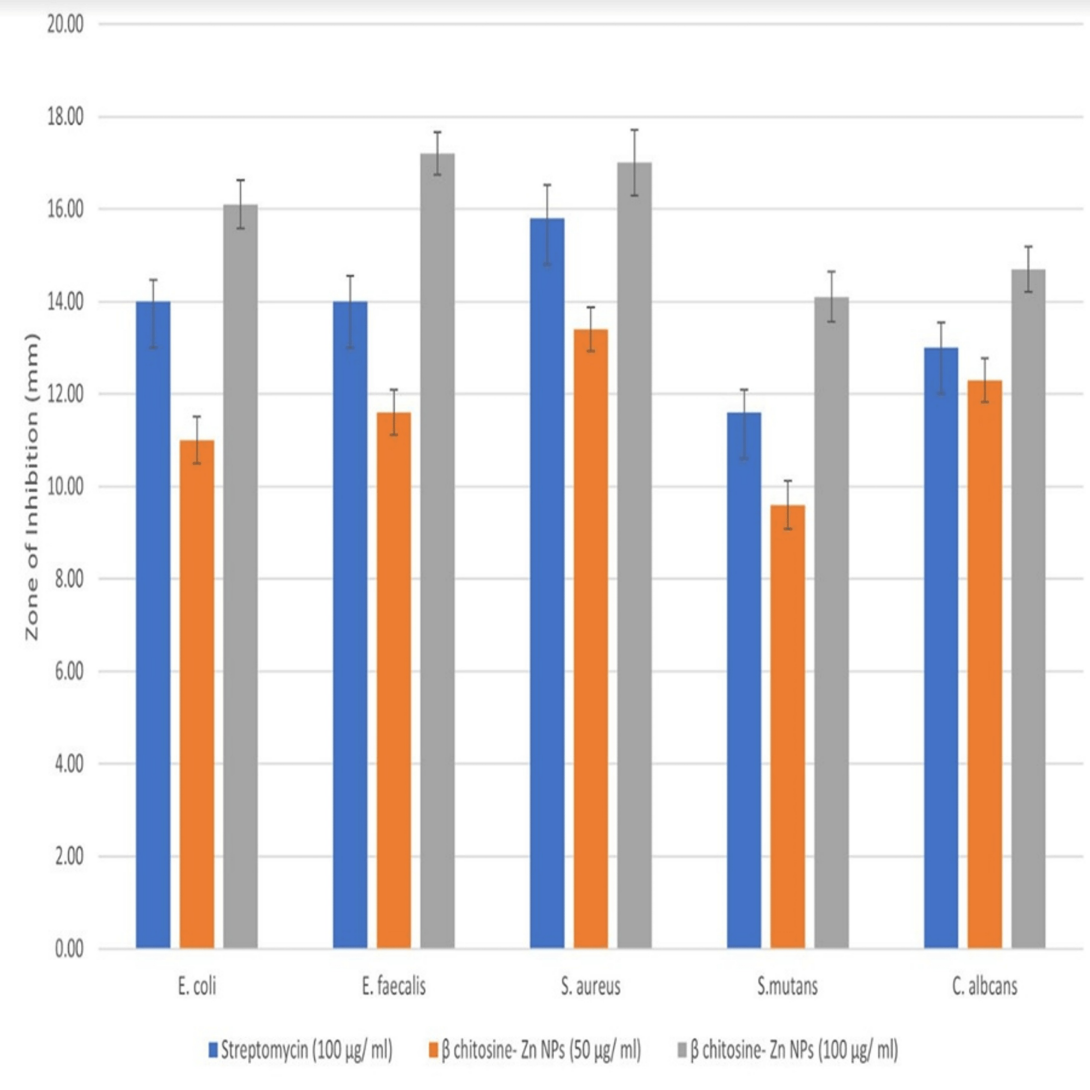
Antimicrobial activity of β-Chitosan-derived zinc oxide nanoparticles against different pathogens. Image credit : Meenakshi Sundaram.

**Figure 4:**
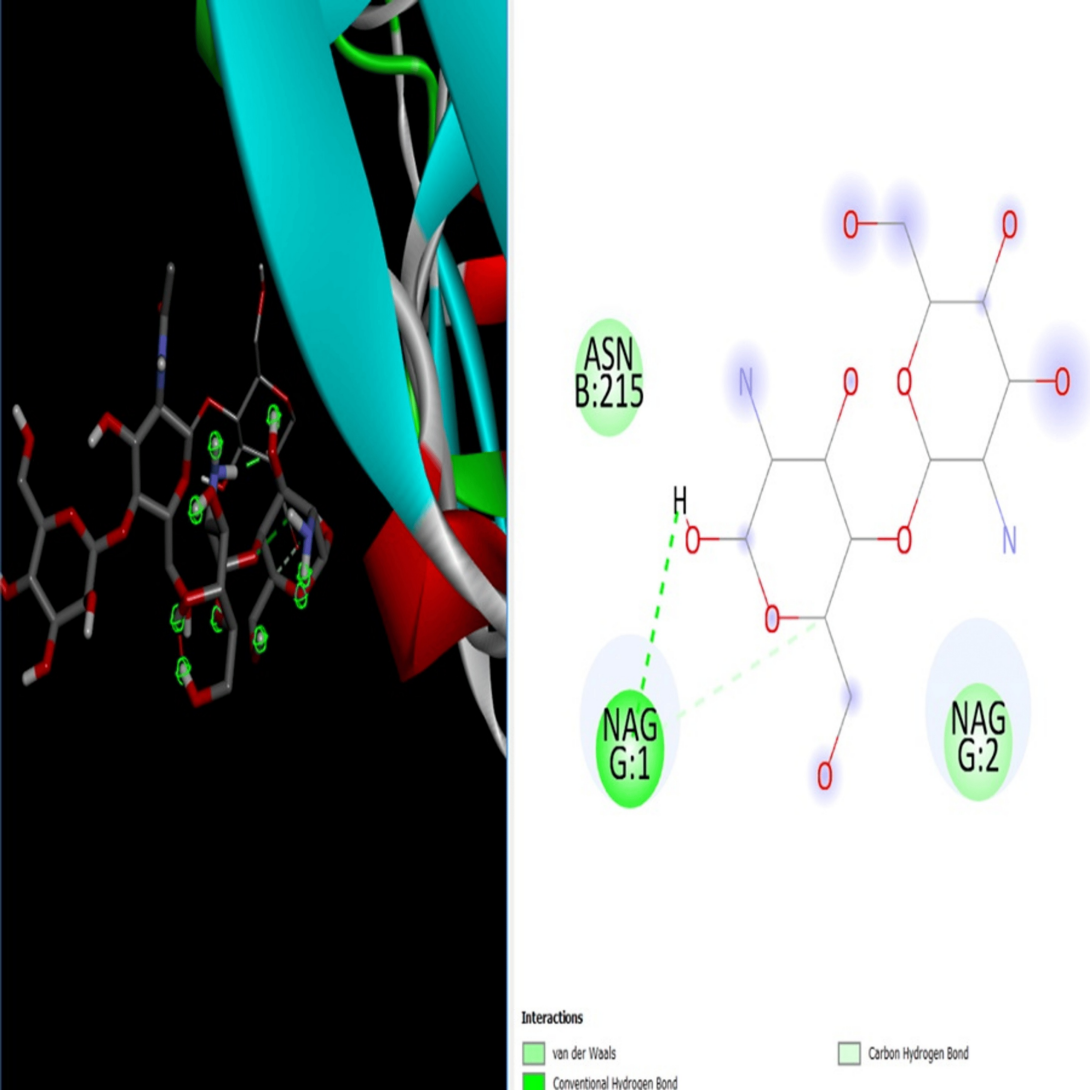
Molecular docking study of receptor, ligand (β-Chitosan) best docking pose and various β-Chitosan-derived zinc oxide nanoparticles interactions with amino acids contribute to cavity formation. Image credit : Meenakshi Sundaram

**Figure 5:**
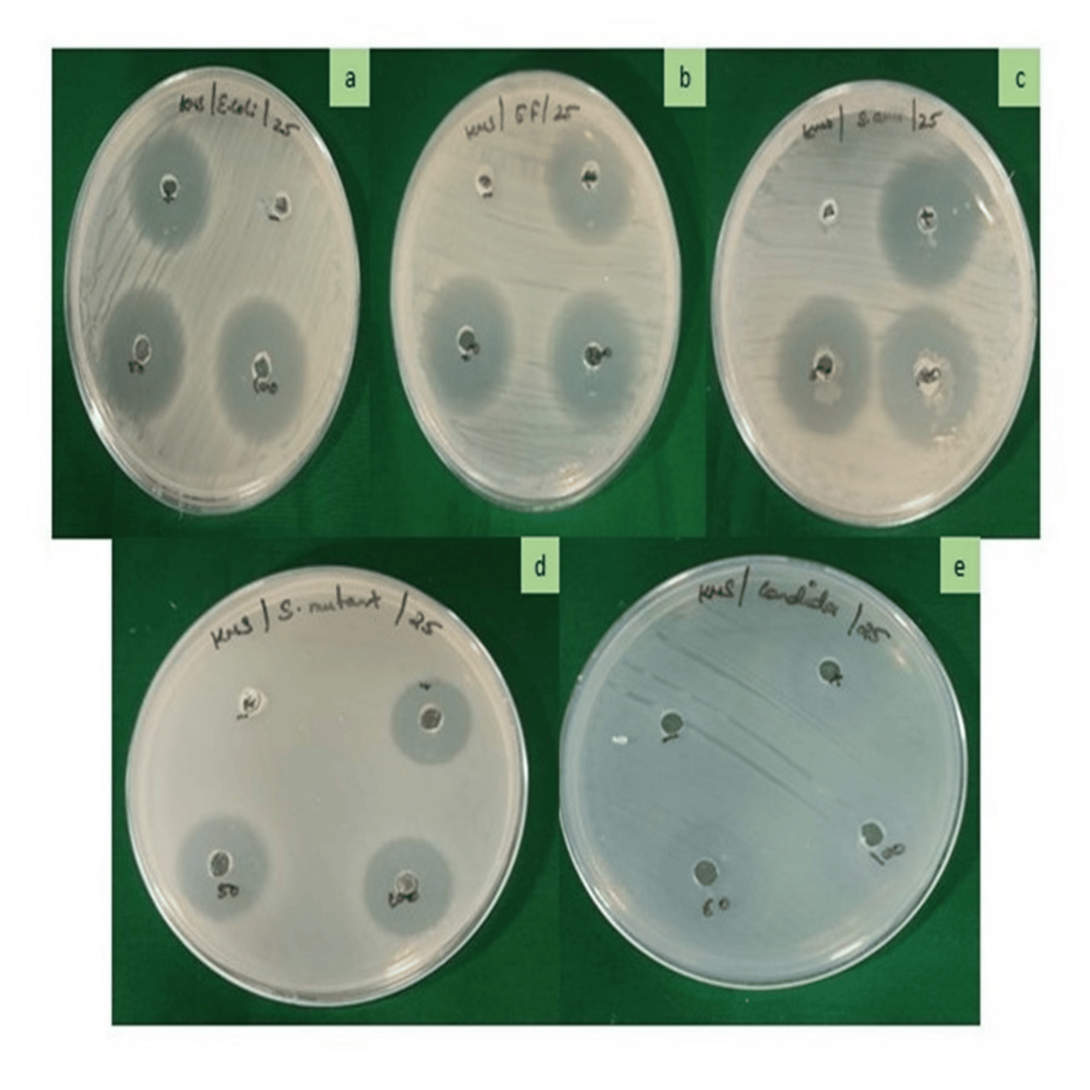
Antimicrobial activity of β-Chitosan-derived zinc oxide nanoparticles for bacterial and fungal strains: (a) Escherichia coli; (b) Enterococcus faecalis; (c) Staphylococcus aureus; (d) Streptococcus mutans; and (e) Candida albicans. Image credit : Meenakshi Sundaram

The table compares the antimicrobial effects of Streptomycin (50 µg/mL) and β-Ch-ZnO-NPs at two different concentrations (50 and 100 µg/mL) on various microorganisms, with the results expressed as the diameter of the inhibition zone in millimeters along with standard deviations (Table 1 and Figure 3). For *E. coli*, Streptomycin shows an inhibition zone of 14 ± 0.46 mm. β-Ch-ZnO-NPs at a concentration of 50 µg/mL have an inhibition zone of 11 ± 0.51 mm, while at 100 µg/mL, the inhibition zone is 16.1 ± 0.52 mm. For *E. faecalis*, Streptomycin has an inhibition zone of 14 ± 0.56 mm, β-Ch-ZnO-NPs at 50 µg/mL show an inhibition zone of 11.6 ± 0.49 mm, and β-Ch-ZnO-NPs at 100 µg/mL demonstrate a larger inhibition zone of 17.2 ± 0.46 mm. For *S. aureus*, Streptomycin exhibits an inhibition zone of 15.8 ± 0.71 mm, β-Ch-ZnO-NPs at 50 µg/mL have an inhibition zone of 13.4 ± 0.48 mm, and β-Ch-ZnO-NPs at 100 µg/mL show an inhibition zone of 17 ± 0.71 mm. For *S. mutans*, Streptomycin shows an inhibition zone of 11.6 ± 0.49 mm, β-Ch-ZnO-NPs at 50 µg/mL have an inhibition zone of 9.6 ± 0.52 mm, and β-Ch-ZnO-NPs at 100 µg/mL demonstrate an inhibition zone of 14.1 ± 0.54 mm. For *C. albicans*, Streptomycin has an inhibition zone of 13.01 ± 0.54 mm, β-Ch-ZnO-NPs at 50 µg/mL show an inhibition zone of 12.3 ± 0.47 mm, and β-Ch-ZnO-NPs at 100 µg/mL show an inhibition zone of 14.7 ± 0.49 mm (Figure 4). In summary, β-Ch-ZnO-NPs at a higher concentration (100 µg/mL) generally exhibit greater antimicrobial activity compared to Streptomycin and the lower concentration of β-Ch-ZnO-NPs (50 µg/mL) across all tested microorganisms (Figure 5).

**Table 1:** Antimicrobial activity of against β-Chitosan-derived zinc oxide nanoparticles against different pathogens.

Microorganism	Streptomycin (50 µg/mL)	β-Ch-ZnO-NPs (50 µg/mL)	β-Ch-ZnO-NPs (100 µg/mL)
* * *Escherichia coli*	14 ± 0.46	11 ± 0.51	16.1 ± 0.52
Enterococcus faecalis	14 ± 0.56	11.6 ± 0.49	17.2 ± 0.46
Staphylococcus aureus	15.8 ± 0.71	13.4 ± 0.48	17 ± 0.71
S. mutans	11.6 ± 0.49	9.6 ± 0.52	14.1 ± 0.54
* * *Candida albicans*	13.01 ± 0.54	12.3 ± 0.47	14.7 ± 0.49

Kruskal-Wallis test

Kruskal-Wallis test was performed to determine if there were statistically significant differences in the antimicrobial activities at different concentrations across the various microorganisms. The test results are summarized in Table 2.

**Table 2:** Results on Kruskal-Wallis test.

Microorganisms	*H*-value	*P*-value
Candida albicans	23.12	<0.001
Streptococcus mutans	21.78	<0.001
Enterococcus faecalis	25.36	<0.001
Escherichia coli	22.45	<0.001
Staphylococcus aureus	24.91	<0.001

Post hoc analysis (Mann-Whitney U-test)

Post hoc analysis using the Mann-Whitney U-test was conducted to compare the antimicrobial activities at 50 and 100 μg/mL for each microorganism (Table 3).

**Table 3:** Results from post hoc analysis (Mann-Whitney U-test).

Microorganisms	Comparison	*U*-value	*P*-value
Candida albicans	50 μg/mL vs. 100 μg/mL	5.0	0.002
Streptococcus mutans	50 μg/mL vs. 100 μg/mL	6.0	0.003
Enterococcus faecalis	50 μg/mL vs. 100 μg/mL	4.0	0.001
Escherichia coli	50 μg/mL vs. 100 μg/mL	5.5	0.002
Staphylococcus aureus	50 μg/mL vs. 100 μg/mL	6.5	0.003

The results indicated here significant increase in the zone of inhibition for all microorganisms tested when the concentration was increased from 50 to 100 µg/mL (Table 4). The Kruskal- Wallis test results showed statistically significant differences (*P *< 0.001) for all microorganisms tested. Post hoc analysis using the Mann-Whitney U-test confirmed these differences were significant for each microorganism, with *P*-values lesser than 0.05.

**Table 4:** Summary of Kruskal-Wallis test and post hoc analysis using the Mann-Whitney U-test.

Microorganism	Concentration (µg/mL)	Mean ± SD	Median	*P*-value
E. coli	50	11 ± 0.51	12.3	<0.001
	100	16.1 ± 0.52	14.7	0.002
E. faecalis	50	11.6 ± .49	11.6	<0.001
	100	17.2 ± 0.46	14.1	0.003
S. aureus	50	13.4 ± 0.48	14.0	<0.001
	100	17 ± 0.71	17.2	0.001
S. mutans	50	9.6 ± 0.52	14.0	<0.001
	100	14.1 ± 0.54	16.1	0.002
C. albicans	50	12.3 ± 0.47	15.8	<0.001
	100	14.1 ± 0.54	17.0	0.003

Molecular docking analysis

A catalytic energy site composed of NAG1 and ASN215 is located in the active site of the 2J0Y protein (PDB: 2J0Y). The catalytic activity of this enzyme can be significantly influenced, inhibited, or halted by targeting these amino acid residues. Notably, the active site residues of the β-Ch-ZnO-NPs receptor are conserved across both Gram-positive and Gram-negative bacteria, making the 2J0Y protein a promising therapeutic target for the development of novel and broad-spectrum antimicrobial drugs as selective and nontoxic 2J0Y inhibitors (Figure 4). To predict the in vitro efficiency of β-Ch-ZnO-NPs, a ligand-2J0Y model was used to perform a molecular docking study. This docking analysis aimed to investigate the proper orientation of β-Ch-ZnO-NPs within the 2J0Y receptor and obtain valuable information regarding the active mechanism, including the non-covalent interactions between the receptor's active site and the NPs. Such insights could lead to the development of new drugs for further biological research.

## Discussion

The exploration of β-Ch-ZnO-NPs for combating dental caries highlights their significant potential in enhancing oral health [18]. Dental caries, a persistent problem driven by cariogenic microbes such as *C. albicans, S. mutans, E. faecalis, E. coli, *and *S. aureus *results from biofilm formation on teeth and the subsequent acid production that erodes enamel [19- 21]. The need for innovative antimicrobial strategies is underscored by the increasing resistance of these microbes to conventional treatments. β-Ch-ZnO-NPs offer a novel approach, leveraging the unique properties of both β-Chitosan and zinc NPs [22]. β-Chitosan, derived from chitin, is not only biocompatible and biodegradable but also exhibits inherent antimicrobial activity. These properties make it a suitable matrix for NPs, enhancing their stability and compatibility with biological systems. Zinc oxide NPs, known for their broad-spectrum antimicrobial efficacy, further strengthen this approach. Their ability to generate reactive oxygen species 9ROS0 under UV light is particularly effective in exerting oxidative stress on microbial cells, thereby disrupting their viability [23]. The synthesis and characterization of β-Ch-ZnO NPs involve precise methodologies to ensure optimal particle size, shape, and stability. techniques such as UV-vis spectroscopy and FTIR are essential in confirming the structural integrity and functional groups of these NPs [24]. The size and morphology of the NPs are crucial, as smaller particles with larger surface areas facilitate better interaction with bacterial cells and biofilms. This enhanced interaction is pivotal in targeting and disrupting biofilms, a critical aspect in the progression of dental caries. The combination of β-Chitosan and zinc oxide NPs aims to synergize their strengths, potentially surpassing the limitations of traditional therapies. β-Chitosan’s biofilm-targeting capabilities, coupled with the potent antimicrobial growth and biofilm formation within the oral cavity. This synergy is expected to lead to more effective prevention and treatment of dental caries [25].

Moreover, the biocompatibility enhancements attributed to β-Chitosan, reduce the cytotoxic effects typically associated with NPs, making β-Ch-ZnO-NPs suitable for various biomedical applications. These include medical implants, drug delivery systems, and tissue engineering aimed at oral health interventions [26]. Functionalizing β-Ch-ZnO-NPs with targeting ligands could further enhance their specificity and efficacy, enabling targeted therapy against cariogenic microorganisms. The unique combination of β-Chitosan's biocompatibility, biodegradability, and antimicrobial properties with the robust antimicrobial efficacy of zinc oxide NPs offers a synergetic approach to addressing the persistent challenge of cariogenic microorganisms [27]. Characterization studies confirm the structural integrity and functional efficacy of β-Ch-ZnO-NPs, with smaller NPs demonstrating enhanced interaction with bacterial cells and biofilms. The biocompatibility improvements attributed to β-Chitosan further expand the potential applications of these NPs in medical implants, drug delivery systems, and tissue engineering [28]. The current study confirms the earlier reports that the combination of β-Chitosan and Zinc NPs could potentiate antimicrobial efficacy. This synergistic effect could be due to the combined mechanism of action, where zinc oxide NPs disturb the microbial cell membranes and β-Chitosan inhibits microbial growth through its bioactive nature. Remmiya et al. reported that ZnO NPs exhibited substantial anti-bacterial effects, particularly at higher concentrations against various microorganisms [25]. Rashki et al. found that β-Chitosan bioactive compound showed potent anti-microbial effects with a dose-dependent relationship [26]. The enhanced antimicrobial activity at a higher concentration at 100 µg/mL could be attributed to the synergistic effect of combining ZnO NPs with β-Chitosan. ZnO NPs are known to disturb the microbial cell membranes and generate reactive oxygen species (ROS) which lead to cell death [29-30]. Meanwhile,β-Chitosan inhibits microbial growth by interfering with microbial metabolism and enzyme functions [25-28]. Molecular docking studies provide deeper insights into the interaction mechanisms of β-Ch-ZnO-NPs with bacterial proteins, elucidating their potential to inhibit critical pathways in microbial metabolism. This understanding paves the way for the development of innovative and broad-spectrum antimicrobial drugs, addressing the issue of bacterial resistance to conventional treatments [22-28].

Despite the promising results highlighted in the exploration of β-Ch-ZnO-NPs for combating dental caries, several limitations must be acknowledged. First, while the antimicrobial efficacy of these NPs appears significant, the study often lacks a comprehensive evaluation of long-term stability and potential side effects in clinical settings. The in vitro findings may not fully translate to in vivo experiments where factors such as saliva flow, pH variations, and complex microbial interactions come into play. Additionally, the synthesis of β-Ch-ZnO-NPs requires precise control over particle size and distribution, which can be challenging to maintain consistently, potentially impacting their effectiveness. The potential for cytotoxicity, despite the biocompatibility of β-Chitosan, needs thorough investigation through long-term safety studies and clinical trials. Thus, translating these findings into practical, widely applicable treatments involves addressing these limitations through rigorous and comprehensive research.

## Conclusions

In conclusion, β-Ch-ZnO-NPs exhibit significant promise as a novel therapeutic approach for preventing and treating dental caries. The multifaceted action, targeting both biofilm formation and microbial growth, combined with enhanced biocompatibility, positions them as a superior alternative to traditional antimicrobial agents. Further research and development could lead to the integration of β-Ch-ZnO-NPs into mainstream dental care, offering improved outcomes in the management of oral health.
